# Co-Community Network Analysis Reveals Alterations in Brain Networks in Alzheimer’s Disease †

**DOI:** 10.3390/brainsci15050517

**Published:** 2025-05-18

**Authors:** Xiaodong Wang, Zhaokai Zhang, Lingli Deng, Jiyang Dong

**Affiliations:** 1 School of Information Science & Technology, Xiamen University Tan Kah Kee College, Zhangzhou 363105, China; wangxd@xujc.com; 2 School of Electronic Science and Engineering (National Model Microelectronics College), Xiamen University, Xiamen 361100, China; 33320221150334@stu.xmu.edu.cn; 3 Department of Information Engineering, East China University of Technology, Nanchang 330013, China; lldeng@ecut.edu.cn

**Keywords:** resting-state fMRI, functional connectivity, Alzheimer’s disease, brain networks, community detection, network physiology

## Abstract

**Background**: Alzheimer’s disease (AD) is a common neurodegenerative disease. Functional magnetic resonance imaging (fMRI) can be used to measure the temporal correlation of blood-oxygen-level-dependent (BOLD) signals in the brain to assess the brain’s intrinsic connectivity and capture dynamic changes in the brain. In this study, our research goal is to investigate how the brain network structure, as measured by resting-state fMRI, differs across distinct physiological states. **Method:** With the research goal of addressing the limitations of BOLD signal-based brain networks constructed using Pearson correlation coefficients, individual brain networks and community detection are used to study the brain networks based on co-community probability matrices (CCPMs). We used CCPMs and enrichment analysis to compare differences in brain network topological characteristics among three typical brain states. **Result:** The experimental results indicate that AD patients with increasing disease severity levels will experience the isolation of brain networks and alterations in the topological characteristics of brain networks, such as the Somatomotor Network (SMN), dorsal attention network (DAN), and Default Mode Network (DMN). **Conclusion:** This work suggests that using different data-driven methods based on CCPMs to study alterations in the topological characteristics of brain networks would provide better information complementarity, which can provide a novel analytical perspective for AD progression and a new direction for the extraction of neuro-biomarkers in the early diagnosis of AD.

## 1. Introduction

The human brain constitutes a highly organized neurobiological system comprising spatially segregated yet functionally integrated regions. Each brain region has its own mission and function. There are biological connections between different brain regions, which are called structural connections. With the development of functional magnetic resonance imaging (fMRI) technology, research on the functional connections between different brain regions has gradually attracted the interest of many researchers. The functional connections between different brain regions, also referred to as the brain’s topological structure, jointly shape complex behaviors [1,2]. The complex network topology of the brain is better suited for analysis from the perspective of network physiology. Network physiology aims to understand how physiological states and functions emerge from the physiological network interactions across physiological systems and subsystems. The scope of network physiology extends far beyond applying knowledge from one field (statistical physics, applied mathematics, informatics, and network theory) to solve problems in another (systems biology, neuroscience, physiology, and medicine). In recent years, we have witnessed the broad impact of introducing novel concepts and methods derived from modern statistical physics and network theory to biology and medicine [3,4]. For example, Miller et al. [5] discovered a special structural organization of neural connections, which allows individual neurons and brain regions to interact as a functional whole.

The majority of functional magnetic resonance imaging (fMRI) techniques utilize paramagnetic deoxyhemoglobin as an endogenous contrast agent. As early as in the 1990s, researchers discovered that blood-oxygenation-level-dependent (BOLD) signal variations could be used to detect brain region activation [6,7]. In fMRI experiments, each scan generates time-series data of BOLD signals across brain regions. Investigations into the correlation of BOLD signals between different brain regions reveal the degree of temporal correlation derived from time-series data, which is called “functional connectivity” (FC). FC is defined as statistically significant temporal dependencies of anatomically separated brain regions. Featuring non-invasive whole-brain imaging capabilities for subjects, fMRI has emerged as a core tool for exploring neural circuit mechanisms underlying major depressive disorder, schizophrenia, and Alzheimer’s disease (AD) [8]. Data analysis using this tool has yielded numerous critical insights for these diseases. However, fMRI demonstrates limited data representation in AD, a neurodegenerative disorder, which restricts its utility in elucidating pathological processes and advancing the mechanistic understanding of AD. Therefore, integrating fMRI with interdisciplinary knowledge to extract multi-dimensional insights and enhance diagnostic capabilities represents a critical frontier in current AD research leveraging this neuroimaging modality. Our research goal is to investigate how the brain network structure differs across distinct physiological states—EMCI, LMCI, and AD. Previous studies have shown that interactions between the brain and peripheral physiological systems also undergo reorganization in neurodegenerative diseases. Prior work has inspired the conceptual framework of this study. For example, Liu et al. [9] observed that brain wave network interactions evolve with the transition from one physiologic state to another following a particular pattern of reorganization. And Bartsch RP et al. determined how organ systems coordinate and optimize their function to produce distinct integrating organ-to-organ interactions into a physiologic network [10].

Graph theory-based complex brain network analysis techniques can be applied to fMRI data to construct brain networks by calculating the functional connectivity between brain regions [11]. Graph theory approaches primarily investigate relationships between nodes and edges. In this context, correlation coefficient calculation refers to quantifying BOLD signal correlations between brain regions, transforming resting-state fMRI data into functional networks composed of nodes and edges amenable to graph analysis. In 2005, Salvador first partitioned the brain into 90 regions using the Automated Anatomical Labeling (AAL) atlas with resting-state fMRI data [12]. By combining resting-state fMRI and graph theory, brain regions can be abstracted as nodes in a network. This approach enables the analysis of interactions between brain regions through correlation coefficients, with both the magnitude and temporal dynamics of these coefficients reflecting changes in functional connectivity—a key focus of fMRI data analysis. Functional connections within brain networks are considered the functional basis for advanced cognitive functions and neurodegenerative diseases. Thus, correlation coefficient calculation not only accurately captures functional associations between brain regions but also plays a pivotal role in brain disease research.

While graph theory provides a powerful statistical tool for analyzing correlations between brain regions via correlation coefficients, its application to fMRI data requires binary thresholding of brain networks—a process that inherently loses raw signal information [13]. Additionally, spatiotemporal data resolution (spatial parcellation and temporal sampling) and inter-subject variability introduce noise into the acquired data, compromising the stability and accuracy of functional connectivity measurements. These limitations reduce the reliability of correlation-based methods in detecting disease-specific connectivity changes and functional modules. Therefore, there is a critical need for novel approaches that minimize inter-subject variability while preserving group differences, enabling the precise identification of disease-related connectivity alterations and associated functional module networks.

Building on Huang’s demonstration of the feasibility of individual brain networks for deriving network properties and distinguishing disease groups [14], our study integrated resting-state fMRI data with graph theory by introducing individual brain networks. By defining differences between disease and cognitively normal groups as constraints in fMRI data, this method constructs individual brain networks that minimize inter-subject variability while preserving group differences and effectively reducing noise in fMRI signals. Leveraging the widely accepted Yeo 7-network parcellation [15], which is a partitioning scheme that divides the entire brain into seven functional networks, including the Visual Network (VIS), Somatomotor Network (SMN), dorsal attention network (DAN), Ventral Attention Network (VAN), Limbic Network (LN), Frontoparietal Network (FN), and Default Mode Network (DMN), our study analyzed alterations in these functional networks during disease progression. In addition, Lee et al. [16] calculated the overlap score, which quantifies the degree of overlap between communities. They revealed that brain networks exhibited significantly higher overlap scores than both their null networks and other real-world networks. Additionally, they showed that brain regions with a high degree of overlap—including the superior frontal gyrus, precuneus, putamen, and thalamus—are located in either the fronto-parietal or subcortical region and are closely associated with cognitive flexibility, a core dimension of executive functions. So, our study innovatively introduced co-community probability into individual brain networks. By combining individual brain networks’ constructions with community detection algorithms, we replaced conventional correlation coefficients with co-community probability to quantify functional connectivity. Statistical testing and enrichment analysis of these matrices enabled the precise identification of alterations in the topological characteristics of brain functional networks. This framework provides a novel perspective on how disease severity impacts functional connectivity patterns within the Yeo 7-network parcellation in Alzheimer’s disease, potentially uncovering new therapeutic targets for early intervention.

## 2. Materials and Methods

### 2.1. Participants

In this study, the dataset was obtained from the Alzheimer’s Disease Neuroimaging Initiative (ADNI) [17], with approval from institutional review boards and informed consent obtained from all participants. The ADNI database has been widely used for the early tracking and detection of AD, aiming to develop biomarkers for the disorder, advance the understanding of AD pathophysiology, and improve diagnostic methods for early detection. Since its launch in 2004, the project has completed three research phases, each involving the recruitment of patients from North America for imaging and clinical assessments. Over time, follow-up imaging data and clinical reassessments were periodically collected to track disease progression. To date, this effort has amassed substantial resting-state fMRI data across diverse patient populations. In this study, the data include 182 resting-state fMRI subjects, consisting of 88 female and 94 male participants. Participants in the dataset were sitting and instructed to keep their eyes open during the fMRI acquisition. Patients were assigned to diagnostic groups categorized as follows: 20 cognitively normal (CN), 57 early mild cognitive impairment (EMCI), 55 late mild cognitive impairment (LMCI), and 50 Alzheimer’s disease (AD) subjects aged 60–80 years. We stratified each disease category by age into three groups: 60s group (aged 60–69), 70s group (aged 70–79), and 80s group (aged 80–89). This resulted in nine disease-specific age groups and one cognitively normal group.

### 2.2. Data Acquisition

Functional images were obtained from the ADNI database with 3 T field strength using Philips Medical Systems scanners, with a matrix size of 64 × 64, a voxel size of 3.3 × 3.3 × 3.3 mm^3^, a GR pulse sequence, 48/36 slices per subject with a 3.3 mm gap (default interleaved slice order), 140 volumes, a TE of 30.0 ms, and a TR of 3001.0 ms.

Structural images were obtained from the ADNI database with 3 T field strength using Philips Medical Systems scanners, with a matrix size of 256 × 256, a GR pulse sequence, a slice gap of 1.2 mm, a TE of 3.1 ms, a TR of 6.8 ms, and a TI of 0.0 ms.

### 2.3. Preprocessing

Preprocessing of datasets included the following steps: (1) reorientation, where distorted images were checked and excluded, and then the origin of remaining fMRI data was reset; (2) slice-timing correction, where top-down inter-leaved slice timing correction was applied to address temporal discrepancies; (3) head motion correction, where we corrected for head movements in six directions (x/y/z translation and rotation); (4) coregistration, where we aligned T1-weighted structural images with functional images; (5) segmentation, where we segmented coregistered structural images into gray matter, white matter, CSF, bone, and others; (6) normalization, which allowed for transformation to the MNI space with 1 mm structural and 3 mm functional resampling; and (7) smoothing, where we applied 6 × 6 × 6 Gaussian kernel smoothing to enhance SNR. All steps were implemented using SPM12 [18] (http://www.fil.ion.ucl.ac.uk/spm (accessed on 4 May 2023)).

The preprocessed resting-state fMRI sample data matrix $X_{91\times 109\times 91\times 140}$ was generated, where the dimensions represent 91 × 109 × 91 spatial voxels and 140 time points. The fMRI data were acquired with a repetition time (TR) of 3 s, resulting in 140 sequential whole-brain scans. The 91 × 109 × 91 dimensions correspond to the spatial volume and anatomical location of the scanned brain.

### 2.4. Computing Individual Brain Network Matrix

Before computing the individual brain network matrix, the functional connectivity matrix was computed using a conventional approach. This calculation was based on the Automated Anatomical Labeling (AAL) atlas proposed by Tzourio-Mazoyer [19], which originally contains 116 brain regions. After excluding 26 cerebellar regions irrelevant to this study, the analysis was performed using the atlas’s remaining 90 cerebral regions. The BOLD signal intensity for each AAL-defined region was calculated as the sum of BOLD signals across all voxels within that region. Through this process, the preprocessed fMRI data matrix $X_{91\times 109\times 91\times 140}$ was partitioned into a BOLD time-series matrix $T_{90\times 140}$, where each sample consists of 90 brain regions with 140 time points per region.

Using the BOLD time-series matrix $T_{90\times 140}$ from the *k*-th subjects, the Pearson correlation coefficient between BOLD signals $x_{i}$ and $x_{j}$ of any two brain regions was computed as follows:

(1) $$C^{k}=(c_{ij}^{k})=corr(x_{i},x_{j}),\forall i,j=1,\;2,\;\ldots ,\;N$$

where *N* denotes the number of brain regions. This resulted in a Pearson correlation coefficient matrix $c_{90\times 90}^{k}$ for each subject, which was then used to construct node–edge functional brain connectivity networks.

The individual brain network matrix was constructed based on the Pearson correlation coefficient matrix through the following procedures.

The mean of the brain region correlation matrices across *H* cognitively normal subjects was computed as follows:

(2) $$\bar{C^{H}}=\left(\bar{c_{ij}^{H}}\right)=\frac{1}{H}\sum_{h=1}^{H}c_{ij}^{h},\;\forall h\in \left\{CN\right\},\;\forall i,j=1,\;2,\;\ldots ,\;N$$

where *H* = 20, and the averaged brain region correlation matrix $\bar{C^{H}}$ of cognitively normal subjects was derived.

The differential correlation matrix $D^{k}$ for the *k*-th disease subject relative to the cognitively normal group matrix $\bar{C^{H}}$ was computed as follows:

(3) $$D^{k}=(d_{ij}^{k})=\frac{\left|c_{ij}^{k}-\bar{c_{ij}^{H}}\right|}{\sqrt{\frac{{\left(c_{ij}^{k}\right)}^{2}+{\left(\bar{c_{ij}^{H}}\right)}^{2}}{2}}},\;\;\forall i,j=1,\;2,\;\ldots ,\;N$$

The differential correlation matrix $D^{k}$ was subjected to Fisher’s *z*-transformation to generate the weighted matrix $W^{k}$ for the *k*-th subject in the disease group, defined as follows:

(4) $$W^{k}=(w_{ij}^{k})=1-\frac{exp\left(2\cdot d_{ij}^{k}\right)-1}{exp\left(2\cdot d_{ij}^{k}\right)+1},\;\;\forall i,j=1,\;2,\;\ldots ,\;N$$

Using the weighted matrix $W^{k},$ the individual brain network matrix $E^{k}$ for the k-th subject in the disease group was computed as follows:

(5) $$E^{k}=(e_{ij}^{k})=w_{ij}^{k}\cdot \;\bar{c_{ij}^{H}},\;\;\forall i,j=1,\;2,\;\ldots ,\;N$$

### 2.5. Community Detection Based on the Louvain Algorithm Was Performed to Compute the Co-Community Probability Matrix

After obtaining the individual brain network matrix for each participant, we adopted the functional connectivity matrix processing approach proposed by Yeo to reduce computational complexity [15]. Specifically, we kept the top 10% of correlations based on the individual brain network matrix $E^{k}$ through thresholding, binarized the sparse matrix, and extracted brain graph networks. The Louvain algorithm, one of the most popular modularity optimization methods widely recognized for its speed and performance in community detection [20], was then applied to partition the network into functional modules. This algorithm operates in two iterative phases until modularity no longer increases: (1) Local Node Movement, where nodes are iteratively reassigned to the community maximizing modularity gain, and (2) Network Aggregation, where an aggregated network is constructed and each community becomes a meta-node.

Given the inherent randomness in the Louvain algorithm’s initialization, repeated community detections were performed for each subject. To mitigate this stochasticity, multiple partition results were randomly sampled. As brain networks with higher community overlap are associated with stronger functional relevance, we computed the co-community probability matrix by quantifying the frequency of two brain regions being assigned to the same community across Louvain community detections. This probability reflects functional connectivity between two brain regions.

For the individual brain network matrix $E^{k}$ of the *k*-th subject, let *m* denote the number of non-zero elements. The corresponding individual brain network $G=\left(V,E\right)$ was constructed, where $V=\left\{v_{1},v_{2},\ldots ,v_{90}\right\}$ represents nodes and $E=\left\{e_{1},e_{2},\ldots ,e_{m}\right\}$ represents edges. This network $G=\left(V,E\right)$ was then split into 90 communities $C_{i}$, each corresponding to a node $v_{i}$ ∈ *V(G)*. The modularity of ***G*** after merging community $C_{i}$ (where *i* = 1, 2, …, 90) with all connected communities $C_{j}$ was computed as follows:

(6) $$Q=\frac{1}{2m}\sum_{c}\left[\sum in-\frac{{\left(\sum tot\right)}^{2}}{2m}\right]$$

Community $C_{i}$ was iteratively merged with the community $C_{j}$ that yielded the maximum modularity change Δ*Q* until all communities $C_{i}$ (*i* = 1, 2, …, 90) were consolidated into a single new community.

The process of computing modularity changes and merging communities was iteratively repeated until no further community assignments changed for all nodes $v_{i}$. One Louvain community partition result was generated. This algorithm was run *M* times for each subject. In this study, *M* = 50. The total number of algorithm executions for each sample group was the product of *M* and the number of subjects within that group. For each subject in this study, five partition results were randomly selected from their M outputs, and *f* presented the total number of partition results selected across the entire sample group. In this study, *f* equals the product of the number of subjects and five. A subset of *f* partition results were randomly selected to compute co-community probabilities. For the *k*-th subject, the co-community probability matrix was calculated as follows:

(7) $$P^{k}=(p_{ij}^{k})=\frac{q}{f},\;\;\forall i,j=1,\;2,\;\ldots ,\;N$$

where *q* denotes the number of times brain regions $v_{i}$ and $v_{j}$ were co-assigned to the same community across *f* partition iterations.

In this study, participants were categorized into three distinct age groups, each further partitioned into three disease subgroups based on disease severity levels. This resulted in a total of nine disease groups for which co-community probability matrices were computed.

### 2.6. Graph Theory Metrics and Statistical Analysis

This study focused on two graph theory metrics: modularity and participation coefficient. According to Yeo’s functional parcellation, the brain’s functional network is divided into seven major functional domains. Sporns highlighted that modularity represents a general property of complex biological systems [21], with modules playing potential functional roles across multiple biological domains—from evolution and development to metabolism and information processing. The modular brain network’s potential functions include enabling specialized information processing and complex dynamics. Inspired by this, we analyzed brain networks from a modular perspective using community detection and community overlap to derive novel indices for evaluating functional connectivity, aiming to uncover pathological features overlooked by conventional methods. Consequently, the performance of brain networks in community detection became the focus of graph-theoretical parameter assessment.

In evaluating the community detection results, modularity is a commonly used metric to measure the quality of community partitions [22]. Modularity *Q* is defined as follows:

(8) $$Q=\frac{1}{2m}\sum_{vw}\left[A_{vw}-\frac{k_{v}k_{w}}{2m}\right]\delta \left(c_{v},c_{w}\right)$$

where *m* is the total number of edges in the network, $A_{vw}$ is the adjacency matrix element defined as 1 if nodes *v* and *w* are connected and 0 otherwise, $\delta \left(c_{v},c_{w}\right)$ is an indicator function equal to 1 when *v* and *w* belong to the same community and 0 otherwise, and $k_{v}$ and $k_{w}$ denote the degrees of nodes *v* and *w*.

Modularity quantifies the topological separation between communities. A higher *Q* value indicates tighter connections within communities and sparser connections between communities.

The participation coefficient is a metric used to quantify the degree of node integration within and across communities [23] and is also used to evaluate community partition quality. This modularity-based approach to node role assignment is applicable to both structural and functional networks and can reveal the role of particularly important nodes in maintaining inter-module communication. The participation coefficient of nodes $P_{i}$ is defined as follows:

(9) $$P_{i}=1-\sum_{s=1}^{N_{M}}{\left(\frac{k_{is}}{k_{i}}\right)}^{2}$$

where $k_{is}$ is the number of edges from node *i* to nodes in community *s*, $k_{i}$ is the total degree of node *i*, and $N_{M}$ is the number of communities connected to node *i*.

A node with stronger intra-community connections has a participation coefficient approaching 0, while a node with evenly distributed connections across communities has a coefficient approaching 1. Thus, in well-partitioned networks, nodes exhibit lower average participation coefficients.

In statistical analysis, *t*-tests were conducted to compare co-community probability matrices across disease subgroups of varying severity levels within the same age group (FDR-corrected *p* < 0.05). Among statistically significant functional connections, we selected those demonstrating significant alterations in co-community probability along the increasing disease severity levels (EMCI → LMCI → AD) for further analysis.

The co-community probability matrices demonstrating significant group differences from *t*-tests were partitioned into seven functional networks based on Yeo’s parcellation: the Visual Network (VIS), Somatomotor Network (SMN), dorsal attention network (DAN), Ventral Attention Network (VAN), Limbic Network (LN), Frontoparietal Network (FN), and Default Mode Network (DMN). Fisher’s exact test was employed to identify significantly enriched functional networks among altered functional connections using *p*-values derived from the enrichment analysis.

## 3. Results

### 3.1. Graph Theory Metrics in Individual Brain Networks

**Figure 1:**
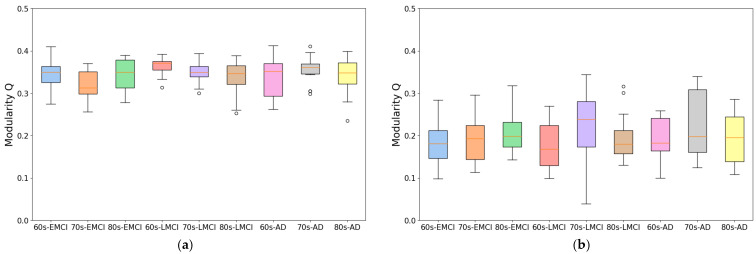
Average modularity in nine disease groups. (**a**) Average modularity using individual brain network method. (**b**) Average modularity using Pearson correlation-based network method.

As shown in Figure 1, we compared the modularity differences between brain networks constructed using two methods in the same participant subjects: a conventional approach based on Pearson correlation coefficients and our proposed individual brain network (IBN) method. The results demonstrate that the IBN method significantly improved modularity across all sample groups. For the IBN method, the average modularities of the nine sample groups all exceeded 0.32, with an overall average modularity of 0.345. In the Pearson correlation-based networks, the average modularities of the nine sample groups all remained below 0.2, with an overall average of 0.199. Specifically, the average modularity of partitioned communities using the IBN method was approximately 70% higher than that of Pearson correlation-based networks.

**Figure 2:**
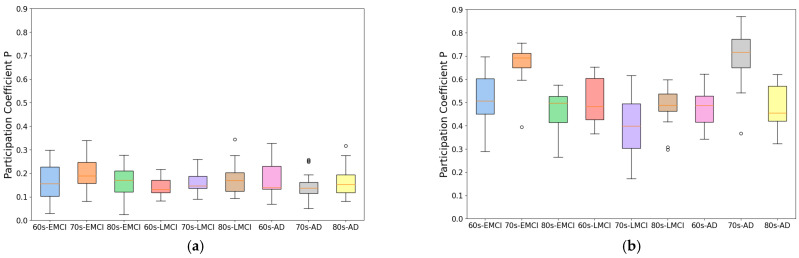
Average participation coefficient in nine disease groups. (**a**) Average participation coefficient using individual brain network method. (**b**) Average participation coefficient using Pearson correlation-based network method.

As shown in Figure 2, we compared the participation coefficient differences between brain networks constructed using Pearson correlation coefficients and our IBN method in the same participant subjects. The results demonstrate that the IBN method significantly reduced participation coefficients across all sample groups. Specifically, for the IBN method, the overall average participation coefficient across the nine sample groups was 0.167; in the Pearson correlation-based networks, this value was 0.521, representing an absolute decrease of 0.354.

An analysis of the two graph theoretical metrics revealed that compared to conventional methods, the IBN method preserved key functional connectivity differences between disease groups and cognitively normal groups while significantly enhancing modularity structure. This improvement in modularity provides a robust data foundation for co-community probability matrix calculations and functional change analyses of brain functional networks.

### 3.2. Co-Community Probability Matrix of Disease Groups

**Figure 3:**
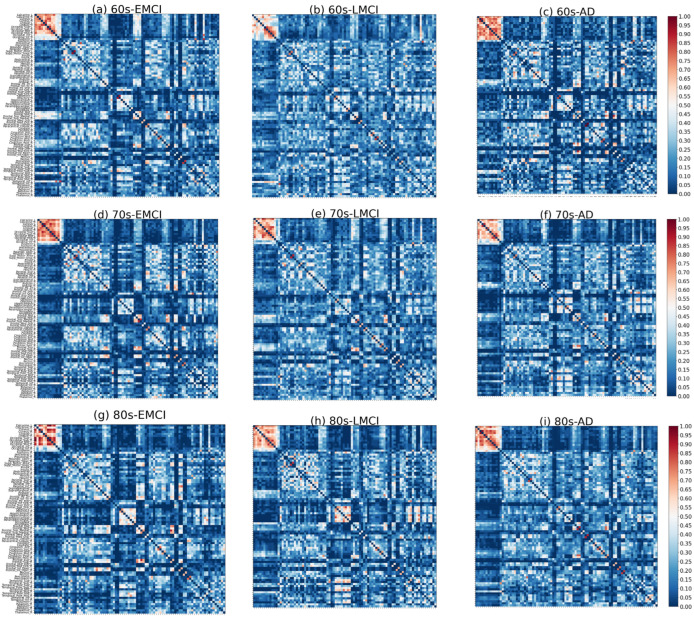
Mean co-community probability matrix of nine disease groups. (**a**–**c**) Three disease subgroups of 60s group. (**d**–**f**) Three disease subgroups of 70s group. (**g**–**i**) Three disease subgroups of 80s group.

**Figure 4:**
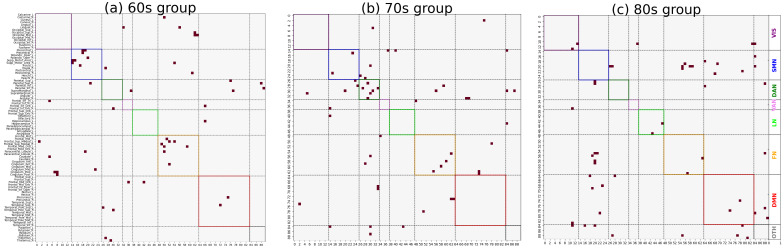
Significantly decreased co-community probability alterations along increasing disease severity levels (EMCI → LMCI → AD) across different age groups within seven functional networks based on Yeo’s parcellation. Values represented in matrix were rearranged by network group according to seven functional networks [VIS, SMN, DAN, VAN, LN, FN, and DMN]. (**a**) Significantly decreased co-community probability alterations in 60s group. (**b**) Significantly decreased co-community probability alterations in 70s group. (**c**) Significantly decreased co-community probability alterations in 80s group.

**Figure 5:**
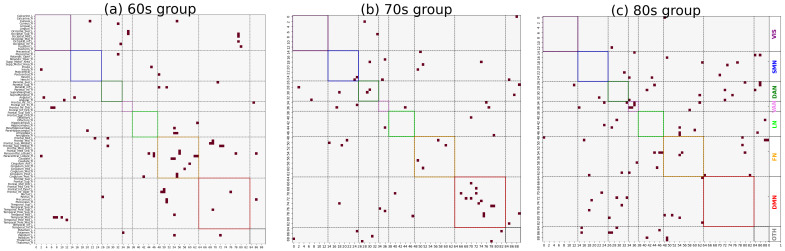
Significantly increased co-community probability alterations along increasing disease severity levels (EMCI → LMCI → AD) across different age groups within seven functional networks based on Yeo’s parcellation. Values represented in matrix were rearranged by network group according to seven functional networks [VIS, SMN, DAN, VAN, LN, FN, and DMN] (**a**) Significantly increased co-community probability alterations in 60s group. (**b**) Significantly increased co-community probability alterations in 70s group. (**c**) Significantly increased co-community probability alterations in 80s group.

Following the previously described procedures, co-community probability matrices between brain regions were computed for each disease group, and the mean co-community probability matrix was calculated as the representative matrix for that group. As shown in Figure 3, significant differences in the distribution of major functional connections were observed across Alzheimer’s disease subgroups of varying severity levels within the same age group. And *t*-tests were conducted to compare co-community probability matrices across disease subgroups of varying severity levels within the same age group (FDR-corrected *p* < 0.05). Among statistically significant functional connections, we selected those demonstrating significant alterations in co-community probability along the increasing disease severity levels (EMCI → LMCI → AD) for further analysis, as presented in Figure 4 and Figure 5.

### 3.3. Fisher’s Exact Test Enrichment Results

As shown in Figure 4 and Figure 5, *t*-tests were conducted to compare co-community probability matrices across disease subgroups of varying severity within the same age group (FDR-corrected *p* < 0.05). Among statistically significant functional connections, those demonstrating significant alterations in co-community probability along the increasing disease severity levels (EMCI → LMCI → AD) were selected. Fisher’s exact test was applied to determine the enrichment of significantly changed co-community probabilities across seven functional networks based on Yeo’s parcellation networks. The enrichment results are presented in Table 1 and Table 2.

## 4. Discussion

Based on the ADNI dataset, this approach introduces co-community probability as an alternative variant to conventional Pearson correlation coefficients to quantify functional connectivity, enhancing the accuracy of detecting pathological connectivity changes and enabling robust subsequent statistical testing and functional enrichment analysis.

To develop this method, we improved traditional correlation-based approaches by introducing an individual brain networks method for fMRI functional connectivity analysis. This method minimizes inter-subject variability while preserving group differences, as demonstrated by its application to Alzheimer’s disease datasets. As shown in Figure 1 and Figure 2, the individual brain network approach achieved a 70% improvement in modularity and a 50% reduction in participation coefficient compared to conventional methods, indicating enhanced network stability and more reliable community detection. This advancement provides a robust framework for analyzing co-community probability and detecting disease-specific connectivity alterations.

As shown in Figure 3 and Figure 4, significant differences in the distribution of major functional connections were observed across Alzheimer’s disease subgroups of varying severity levels within the same age group. And the distribution of significant co-community probability alterations along the increasing disease severity levels (EMCI → LMCI → AD) across seven functional networks based on Yeo’s parcellation was modulated by age, leading to differential enrichment results.

As shown in Table 1 and Table 2, we observed significantly decreased co-community probability alterations, which were enriched within the SMN and FN in the 60s group. Consistent with previous studies in the literature, the reduced connectivity within the SMN may be attributed to disrupted connections involving the postcentral gyrus [24,25], which played a critical role in the somatosensory processing of pain and temperature [26]. Additionally, insular atrophy could contribute to decreased functional connectivity within this network [27]. Within the FN, reduced connectivity may be associated with the frontal lobe [28]. The frontal lobe is responsible for logic, regulating behavior, complex planning, and learning. As the disease progresses, changes in the frontal lobe will lead to loss of motivation and cause apathy in patients.

In the 70s group, significantly decreased co-community probabilities were enriched in the DAN. Conversely, significantly increased co-community probabilities were enriched in the DMN. This finding aligns with Esposito’s observation of spontaneous anticorrelated activity between the DMN and DAN [29]. The underlying mechanism may involve functional anticorrelation as a critical aspect of intrinsic brain functional organization, allowing for the identification of markers indexing the conversion of MCI to AD.

Our data reveal age-specific differences in functional network changes during Alzheimer’s disease (AD) progression, leading us to recommend paying particular attention to the age groups of subjects in AD functional network studies. Our findings emphasize the dynamic alterations in these functional networks during AD progression, complementing our understanding of AD-related brain network dysfunction from the perspective of co-community probability matrix enrichment. The identified functional networks and their observed changes were validated in prior AD research, confirming the reliability of our methodology. Additionally, in the 60s group, significantly increased co-community probabilities were enriched in the FN; in the 80s group, such increases were observed in the VAN. These findings may reflect metabolic compensation or network reorganization within functional networks. Therefore, these functional networks should receive equal attention to the previously described ones in AD pathological studies as they may provide novel insights into AD’s pathological mechanisms. The enrichment analysis results in Table 1 and Table 2 indicate that, in the 60s group, all brain region pairs with increased co-community probabilities were more concentrated in the FN across the whole brain, while those with decreased co-community probabilities were also more enriched in the FN with disease progression. A plausible hypothesis for this phenomenon is that the Frontoparietal Network serves as a functional hub of the brain, and disease-related alterations lead to extensive functional changes within the FN, involving not only decreases but also increases in co-community probabilities.

In conclusion, by introducing co-community probability to replace traditional methods and employing community enrichment analysis, our study identified functional domains consistent with previously reported alterations in AD, collectively explaining the pathological remodeling of intra-network functional relationships in AD patients. This work suggests that using different data-driven methods based on CCPM to study alterations in the topological characteristics of brain networks would have better in-formation complementarity, which can provide a novel analytical perspective for AD progression. However, this study primarily focused on the enrichment of co-community probability changes within functional networks. In contrast, Wang identified dysregulated functional connectivity between the DAN and DMN in patients with cognitive impairment and AD [30]. Buckner further demonstrated age-related disconnected connectivity among the DAN, FN, and DMN [31]. Therefore, the co-community probability relationships between functional networks remained an underexplored area requiring further investigation. In addition, Rizzo had shown that interactions between the brain and peripheral physiological systems also undergo reorganization in neurodegenerative diseases [32]. This work shows that if the co-community probability matrix approach is applied to the dynamic networks of brain–organ interactions in relation to basic physiological states found in network physiology, an enrichment analysis of co-community probabilities could potentially identify novel network-based biomarkers for AD patients.

## Figures and Tables

**Table 1:** Enrichment results of significantly decreased co-community probability along increasing disease severity levels (EMCI → LMCI → AD) across different age groups within seven functional networks based on Yeo’s parcellation.

	60s Group	70s Group	80s Group
Visual Network	*p* = 1.0000	*p* = 1.0000	*p* = 0.4509
Somatomotor Network	*p* = 0.0007	*p* = 0.3408	*p* = 1.0000
Dorsal Attention Network	*p* = 1.0000	*p* = 0.0006	*p* = 1.0000
Ventral Attention Network	*p* = 1.0000	*p* = 1.0000	*p* = 1.0000
Limbic Network	*p* = 1.0000	*p* = 1.0000	*p* = 0.2553
Frontoparietal Network	*p* = 0.0059	*p* = 0.1713	*p* = 0.5477
Default Mode Network	*p* = 1.0000	*p* = 0.6296	*p* = 0.6331

**Table 2:** Enrichment results of significantly increased co-community probability along increasing disease severity levels (EMCI → LMCI → AD) across different age groups within seven functional networks based on Yeo’s parcellation.

	60s Group	70s Group	80s Group
Visual Network	*p* = 1.0000	*p* = 1.0000	*p* = 1.0000
Somatomotor Network	*p* = 1.0000	*p* = 0.3936	*p* = 1.0000
Dorsal Attention Network	*p* = 1.0000	*p* = 0.1904	*p* = 1.0000
Ventral Attention Network	*p* = 1.0000	*p* = 1.0000	*p* = 0.0015
Limbic Network	*p* = 1.0000	*p* = 1.0000	*p* = 1.0000
Frontoparietal Network	*p* = 0.0035	*p* = 0.2263	*p* = 1.0000
Default Mode Network	*p* = 1.0000	*p* = 0.0024	*p* = 0.2645

## Data Availability

Data used in the preparation of this article were obtained from the ADNI database (https://adni.loni.usc.edu/ (accessed on 15 May 2025)). The authors’ data are available upon reasonable request and with the ADNI’s approval.

## References

[1] Park H.J., Friston K. (2013). Structural and functional brain networks: From connections to cognition. Science.

[2] Stam C.J. (2014). Modern network science of neurological disorders. Nat. Rev. Neurosci..

[3] Ivanov P.C. (2021). The New Field of Network Physiology: Building the Human Physiolome. Front. Netw. Physiol..

[4] Ivanov P.C., Liu K.K.L., Bartsch R.P. (2016). Focus on the emerging new fields of Network Physiology and Network Medicine. New J. Phys..

[5] Muller L., Destexhe A., Rudolph-Lilith M. (2014). Brain networks: Small-worlds, after all?. New J. Phys..

[6] Ogawa S., Lee T., Nayak A.S., Glynn P. (1990). Oxygenation-sensitive contrast in magnetic resonance image of rodent brain at high magnetic fields. Magn. Reson. Med..

[7] Kwong K.K., Belliveau J.W., Chesler D.A., Goldberg I.E., Weisskoff R.M., Poncelet B.P., Kennedy D.N., Hoppel B.E., Cohen M.S., Turner R. (1992). Dynamic magnetic resonance imaging of human brain activity during primary sensory stimulation. Proc. Natl. Acad. Sci. USA.

[8] Buckner R.L., Krienen F.M., Yeo B.T.T. (2013). Opportunities and limitations of intrinsic functional connectivity MRI. Nat. Neurosci..

[9] Liu K.K.L., Bartsch R.P., Lin A., Mantegna R.N., Ivanov P.C. (2015). Plasticity of brain wave network interactions and evolution across physiologic states. Front. Neural Circuits.

[10] Bartsch R.P., Liu K.K.L., Bashan A., Ivanov P.C. (2015). Network Physiology: How organ systems dynamically interact. PLoS ONE.

[11] Liang X., Zou Q., He Y., Yang Y. (2013). Coupling of functional connectivity and regional cerebral blood flow reveals a physiological basis for network hubs of the human brain. Proc. Natl. Acad. Sci. USA.

[12] Salvador R., Suckling J., Coleman M.R., Pickard J.D., Menon D., Bullmore E. (2005). Neurophysiological architecture of functional magnetic resonance images of human brain. Cereb. Cortex.

[13] Shahhosseini Y., Miranda M.F. (2022). Functional Connectivity Methods and Their Applications in fMRI Data. Entropy.

[14] Huang S.-Y., Hsu J.-L., Lin K.-J., Hsiao I.-T. (2020). A Novel Individual Metabolic Brain Network for 18F-FDG PET Imaging. Front. Neurosci..

[15] Yeo B.T., Krienen F.M., Sepulcre J., Sabuncu M.R., Lashkari D., Hollinshead M., Roffman J.L., Smoller J.W., Zöllei L., Polimeni J.R. (2011). The organization of the human cerebral cortex estimated by intrinsic functional connectivity. J. Neurophysiol..

[16] Lee B., Kang U., Chang H., Cho K.-H. (2022). The hidden community architecture of human brain networks. Sci. Rep..

[17] Jack C.R., Bernstein M.A., Fox N.C., Thompson P., Alexander G., Harvey D., Borowski B., Britson P.J., Whitwell J.L., Ward C. (2008). The Alzheimer’s Disease Neuroimaging Initiative (ADNI): MRI methods. J. Magn. Reson. Imaging.

[18] Chao-Gan Y., Yu-Feng Z. (2010). DPARSF: A MATLAB Toolbox for “Pipeline” Data Analysis of Resting-State fMRI. Front. Syst. Neurosci..

[19] Tzoutio-Mazoyera N., Landeau B., Papathanassiou D., Crivello F., Etard O., Delcroix N., Tzourio-Mazoyer B., Joliot M. (2002). Automated anatomical labeling of activations in SPM using a macroscopic anatomical parcellation of the MNI MRI single-subject brain. Neuroimage.

[20] Traag V.A., Waltman L., Van Eck N.J. (2019). From Louvain to Leiden: Guaranteeing well-connected communities. Sci. Rep..

[21] Sporns O., Betzel R.F. (2016). Modular Brain Networks. Annu. Rev. Psychol..

[22] Newman M.E. (2006). Modularity and community structure in networks. Proc. Natl. Acad. Sci. USA.

[23] Guimerà R., Amaral L.A. (2005). Cartography of complex networks: Modules and universal roles. J. Stat. Mech..

[24] Wang P., Zhou B., Yao H., Zhan Y., Zhang Z., Cui Y., Xu K., Ma J., Wang L., An N. (2015). Aberrant intra- and inter-network connectivity architectures in Alzheimer’s disease and mild cognitive impairment. Sci. Rep..

[25] Li W., Wen W., Chen X., Ni B., Lin X., Fan W., Initiative T.A.D.N. (2020). The Alzheimer’s Disease Neuroimaging Initiative. Functional Evolving Patterns of Cortical Networks in Progression of Alzheimer’s Disease: A Graph-Based Resting-State fMRI Study. Neural Plast.

[26] Fletcher P.D., Downey L.E., Golden H.L., Clark C.N., Slattery C.F., Paterson R.W., Rohrer J.D., Schott J.M., Rossor M.N., Warren J.D. (2015). Pain and temperature processing in dementia: A clinical and neuroanatomical analysis. Brain.

[27] Sammons R.D., Gaines T.A. (2014). Right anterior insula: Core region of hallucinations in cognitive neurodegenerative diseases. PLoS ONE.

[28] Zhao Q., Lu H., Metmer H., Li W.X., Lu J. (2018). Evaluating functional connectivity of executive control network and frontoparietal network in Alzheimer’s disease. Brain Res..

[29] Esposito R., Cieri F., Chiacchiaretta P., Cera N., Lauriola M., Di Giannantonio M., Tartaro A., Ferretti A. (2018). Modifications in resting state functional anticorrelation between default mode network and dorsal attention network: Comparison among young adults, healthy elders and mild cognitive impairment patients. Brain Imaging Behav..

[30] Wang J., Liu J., Wang Z., Sun P., Li K., Liang P. (2019). Dysfunctional interactions between the default mode network and the dorsal attention network in subtypes of amnestic mild cognitive impairment. Aging.

[31] Buckner R.L. (2004). Memory and executive function in aging and AD: Multiple factors that cause decline and reserve factors that compensate. Neuron.

[32] Sammons R.D., Gaines T.A. (2023). Dynamic networks of cortico-muscular interactions in sleep and neurodegenerative disorders. Front. Netw. Physiol..

